# Distinct immune responses confer partial resistance to Fusarium wilt in tomato landraces

**DOI:** 10.1007/s00425-025-04818-7

**Published:** 2025-09-08

**Authors:** Antonis Tzionis, Giorgos Artymatas, Angelos C. Kyratzis, Stavroula Dimitriadi, Maria-Dimitra Tsolakidou, Iakovos S. Pantelides

**Affiliations:** 1 Department of Agricultural Sciences, Biotechnology and Food Science, Cyprus University of Technology, 3603 Lemesos, Cyprus; 2 Vegetable Crop Sector, Agricultural Research Institute-Ministry of Agriculture, Rural Development and Environment, 1516 Nicosia, Cyprus

**Keywords:** Plant-pathogen interactions, Defense signaling pathways, Solanaceae germplasm, Vascular wilt disease

## Abstract

**Main Conclusion:**

Cypriot tomato landraces exhibit partial resistance to Fusarium wilt through distinct jasmonic and salicylic acid-mediated immune responses, offering promising genetic resources for breeding durable tomato cultivars.

**Abstract:**

Fusarium wilt, caused by *Fusarium oxysporum* f. sp. *lycopersici* (*Fol*), is a major constraint on global tomato (*Solanum lycopersicum*) production, with few sustainable control measures available. This study assessed six Cypriot tomato landraces for resistance to *Fol* and explored the mechanisms underlying their defense. Pathogenicity assays under controlled growth conditions identified two landraces, ARI00732 and ARI00733, with partial resistance and improved growth performance compared to the susceptible cultivar Ailsa Craig. A second pathogenicity trial using sterilized and non-sterilized soils revealed no significant contribution of soil microbiota, suggesting intrinsic plant defenses as the primary mechanism. In vitro assays showed that root exudates from these landraces neither inhibited *Fol* growth nor altered fungal chemotropism. Gene expression analysis revealed distinct defense strategies: ARI00732 displayed strong induction of jasmonic acid (JA)-responsive genes (*MYC2*, *LoxD*, *PDF1.2*), whereas ARI00733 upregulated salicylic acid (SA)-associated *Pti5* gene and the antioxidant defense gene *APX1*. These findings demonstrate that complementary JA- and SA-mediated pathways contribute to resistance. This work highlights the potential of tomato landraces as a source of durable resistance traits and provides a foundation for breeding programs targeting Fusarium wilt.

**Supplementary Information:**

The online version contains supplementary material available at 10.1007/s00425-025-04818-7.

## Introduction

Tomato (*Solanum lycopersicum*) is one of the most important vegetable crops globally, with a significant role in both human nutrition and the economy. It is a rich source of vitamins, minerals and antioxidants, which contribute to its popularity worldwide (Bergougnoux 2014). In Cyprus, tomato cultivation has a long tradition, with the crop being an integral part of the local agricultural landscape (Athinodorou et al. 2021). However, tomato production is increasingly threatened by various pathogens, among which *Fusarium oxysporum* f. sp. *lycopersici* (*Fol*) is particularly notorious (Srinivas et al. 2019).

*Fol* is a soilborne fungal pathogen causing Fusarium wilt, a devastating disease that affects tomato plants (McGovern 2015a). The pathogen is highly specialized and is classified into three main physiological races (races 1, 2, and 3), each capable of infecting different groups of tomato cultivars (Srinivas et al. 2019). *Fol* invades the plant primarily through the roots, colonizing the vascular system and obstructing water and nutrient flow. This leads to characteristic symptoms such as yellowing of leaves, wilting, stunted growth and eventually, plant death (Olivain and Alabouvette 1999). The pathogen's ability to survive in soil for extended periods, often in the form of chlamydospores, makes it particularly difficult to manage. This persistence in the soil, coupled with its ability to infect plants without overt symptoms until the disease is advanced, poses a substantial challenge to tomato growers worldwide (Yadeta and Thomma 2013; Antoniou et al. 2017; Srinivas et al. 2019). Fusarium wilt is a significant constraint on tomato production, not only because of the direct losses it causes but also due to the increased costs associated with its management. The yield loss caused by the disease may range from 30 to 40%, and may reach 80% under favorable weather conditions (Nirmaladevi et al. 2016).

Over the years, different methods have been developed to control Fusarium wilt in tomato plants with different levels of success. The primary methods include cultural practices, chemical control, biological control and the use of resistant tomato varieties. However, each of these approaches has significant limitations.

Cultural practices such as crop rotation, soil solarization, and the use of pathogen-free seedlings are commonly employed to manage Fusarium wilt in tomatoes. Crop rotation, which involves alternating tomato crops with non-host plants, can help reduce pathogen buildup (De Corato et al. 2020); however, its effectiveness is limited by the long survival of *Fusarium oxysporum* in the soil, which can persist for years without a host (Bailey and Lazarovits 2003). Soil solarization can effectively lower *Fusarium* populations by raising soil temperatures, but its use is often hindered by environmental conditions and large-scale application challenges (Abed Gatea Al-Shammary et al. 2020). Moreover, cultural practices alone are insufficient for controlling Fusarium wilt, particularly in regions where the pathogen is well-established (Ajilogba and Babalola 2013). Integrated approaches, including the use of plant growth-promoting microorganisms and biocontrol agents, have shown promise in enhancing disease resistance and improving tomato yields (Antoniou et al. 2017; Tsolakidou et al. 2019; Attia et al. 2022).

Chemical control has traditionally been a key component of Fusarium wilt management. Fungicides, particularly soil fumigants, have been employed to reduce *Fol* populations in the soil and protect tomato plants from infection. However, the effectiveness of chemical control methods has become increasingly limited due to several factors. One of the most significant setbacks was the ban of methyl bromide in 2005, which was widely used as a soil fumigant and proved effective against the resting structures of *Fol*. The ban on methyl bromide and other chemical fumigants was enacted due to serious public health and environmental concerns, as these chemicals can have harmful effects on non-target organisms, including beneficial soil microbes (Pascale et al. 2020). Additionally, the efficacy of pesticides is often compromised because they are unable to penetrate the soil matrix effectively, which limits their reach and impact (De Coninck et al. 2015). Moreover, there are currently no effective chemical treatments available to cure plants once they are infected with *Fol* (Antoniou et al. 2017).

The development and use of resistant tomato varieties have been the most effective strategy for managing Fusarium wilt. Breeding programs worldwide have focused on incorporating resistance genes (e.g., *I*, *I-2*, and *I-3*) into commercial tomato varieties (Chitwood-Brown et al. 2021). These genes confer resistance to specific races of *Fol*, offering a level of protection that cultural and chemical controls cannot achieve. However, the effectiveness of resistant varieties is challenged by the rapid evolution of *Fol*. The emergence of new physiological races, such as race 3, has led to the breakdown of resistance in previously resistant cultivars (Swett et al. 2023). This"arms race"between pathogen evolution and breeding efforts necessitates a continuous search for new sources of resistance.

Given the limitations of existing management strategies, there is an increasing interest in exploring the potential of local tomato varieties as sources of resistance to Fusarium wilt. Local varieties, often referred to as landraces, are locally adapted populations that have been cultivated for generations by farmers. Their evolution in time and space was mediated by farmers selections and natural forces. These varieties are typically characterized by a high degree of genetic diversity, which can confer resilience to various biotic and abiotic stresses, including Fusarium wilt of tomato (Sultan et al. 2024).

In the context of Cyprus, the local tomato populations represent a valuable but underutilized genetic resource. These populations have evolved under the specific environmental conditions of the island, which include a Mediterranean climate with hot, dry summers and mild, wet winters. The adaptation of these varieties to local conditions may have endowed them with unique traits, including resistance to locally prevalent pathogens such as *Fol*. Unlike commercial varieties, which are often bred for uniformity and market traits, local varieties maintain a high level of genetic diversity, which can provide a broader spectrum of resistance to pathogens and adjustment to climate change (Blanca et al. 2015; Khoury et al. 2022).

The potential of local tomato populations of Cyprus to resist Fusarium wilt is of particular interest because it offers a sustainable approach to managing the disease. By identifying and utilizing resistant varieties, it is possible to reduce reliance on chemical control, decrease the environmental impact of tomato cultivation, and enhance the resilience of local agricultural systems.

The present study aims to investigate the potential resistance of six tomato landraces from Cyprus against *Fol*. By evaluating these populations for their resistance to Fusarium wilt, this research seeks to identify candidates for breeding programs aiming to develop Fusarium-resistant tomato varieties. The findings from this study could significantly support sustainable tomato production in Cyprus and other regions with similar environmental conditions, contributing to reduced chemical input, enhanced crop resilience and the preservation of local biodiversity.

## Materials and methods

### Plant material and growth conditions

The plant material used in this study included six distinct Cypriot tomato landraces: ARI00732, ARI00733, ARI00734, ARI00735, ARI00738, and ARI00739, which are conserved in the genebank of the Agricultural Research Institute (ARI) in Cyprus. These accessions were evaluated for their resistance to *Fol*. In addition to these landraces, tomato cultivars Ailsa Craig (Thompson & Morgan Ltd.) and Alliance F1 (Clause vegetable seeds) were also used in the experiments. Ailsa Craig, known for its susceptibility to *Fol*, served as the positive control, while Alliance F1, which is resistant to *Fol*, served as the negative control.

Tomato seeds were surface sterilized according to the method described by Chialva et al. (2016). The sterilized seeds were then sown in square pots measuring 9 cm × 9 cm × 8 cm (Pöppelmann TEKU VQB 9 × 9 × 8), each containing approximately 500 cm^3^ substrate composed of potting mix (Plantobalt substrate 2, Plantaflor, Vechta, Germany) and perlite in 3:1 ratio (Antoniou et al. 2017). The plants were grown in a controlled growth chamber maintained at 25 °C with a 16-h light and 8-h dark cycle, 65–70% relative humidity, and a light intensity of 450 μmol m^−2^ s^−1^ at pot level. The plants were irrigated every other day, ensuring each received the same amount of water.

### Fungal strain and inoculum preparation

The *Fol *race 1 isolate (Fol004; Rep et al. 2005), used in this study was sourced from the culture collection at the Department of Agricultural Sciences, Biotechnology, and Food Science at Cyprus University of Technology, Limassol, Cyprus. The fungal spores were suspended in a 20% glycerol solution and cryopreserved at −80 °C. Before use, the fungal strain was revived on potato dextrose agar (PDA, Merck, Rahway, NJ, USA) and incubated at 25 °C for 5 days. For the pathogenicity assays, conidia were prepared by transferring fragments from the actively growing edge of the fungal colony into sucrose sodium nitrate (SSN) medium (Sinha and Wood 1968) within 500 mL Erlenmeyer flasks. The flasks were then incubated on an orbital shaker (150 rpm) in the dark at 25 °C for 5 days. The liquid cultures were filtered through cheesecloth to remove mycelia, followed by centrifugation at 10,000×*g* at 8 °C for 10 min. The resulting pellets were resuspended in sterile distilled water (SDW). The concentration of conidia in the suspension was quantified using a hemocytometer to ensure accurate spore counts before inoculation. The suspensions were then diluted with sterile distilled water to achieve a final concentration of 10^7^ conidia/mL.

### Pathogenicity experiments

The resistance of the six Cypriot landraces against *Fol* was evaluated through pathogenicity experiments conducted in two separate growth room trials.

In the first trial, the six landraces were evaluated against *Fol* under controlled growth room conditions. The plants were inoculated at the third leaf stage (approximately 20 days after seedling emergence) by root drenching with 20 mL of a conidial suspension of *Fol*. This inoculation method involved applying the suspension directly to the root zone of the plants in their pots. Control plants were mock-inoculated with 20 mL of sterile distilled water. The plants were arranged in a completely randomized design and rotated within the growth room every two days before watering. Disease severity was assessed by counting the number of leaves exhibiting typical symptoms (wilting, yellowing, and browning) as a percentage of the total leaf count per plant. Symptom development was recorded periodically for 22 days post-inoculation (dpi). The disease progression over time was plotted to create disease progress curves, and the area under the disease progress curve (AUDPC) was calculated using the trapezoidal integration method (Campbell and Madden 1990). The disease was expressed as a percentage of the maximum possible area for the entire duration of the experiment, referred to as relative AUDPC. In addition to disease assessments, plant growth parameters were measured at the end of the experiment. Plant fresh weight was determined by carefully cutting the plant at the base of the stem, excluding the roots, and weighing the above-ground portion using a precision balance. Plant height was measured from the base of the stem to the apex of the longest leaf using a ruler. The total number of leaves per plant, including senesced or fully necrotic leaves, was counted to assess overall leaf production. These measurements were used to evaluate the impact of *Fol* infection on plant growth. This experiment included twelve replicates for each treatment.

Based on the results of the first trial, which identified ARI00732 and ARI00733 as having the highest resistance to *Fol*, a second experiment was conducted to determine whether this resistance was influenced by beneficial microorganisms in the soil or was solely due to the genetic traits of the plants. In this experiment, the plants were grown under the same conditions as in the first trial but they were planted in either sterilized or unsterilized soil. The substrate was sterilized by steam (100 °C for 1 h, repeated over three consecutive days) to eliminate all microorganisms (Antoniou et al. 2017), while unsterilized soil was used as a control. The sterilized substrate was stored in a laminar flow unit at room temperature between sterilization cycles. Disease severity, AUDPC, and plant growth parameters (fresh weight and height) were assessed as described for the first trial. Total leaf area was determined from digital images of the plants using ImageJ software (Schneider et al. 2012). This experiment included twelve replicates for each treatment.

### Fungal quantification in planta

The level of fungal colonization in the vascular tissues of tomato plants was determined by real-time quantitative PCR (qPCR). At 23 days post-inoculation (dpi), twelve plants from each treatment group were harvested. For each biological replicate (a pool of four plants per treatment), the above-ground plant parts were collected, discarding the leaves, and the stems were ground to a fine powder with a mortar and pestle in liquid nitrogen. Total DNA was extracted following the protocol of Dellaporta et al. (1983), quantified using a NanoDrop 2000 spectrophotometer (Thermo Scientific), and adjusted to a concentration of 100 ng/μl. qPCR was performed using the StepOnePlus™ Real-Time PCR System (Thermo Fisher Scientific) and the KAPA SYBR FAST qPCR Master Mix (KAPA Biosystems, Inc., Boston, MA, USA).

The primer pair sp1-2f and sp1-2r (Inami et al. 2010) which targets the rDNA-intergenic spacer region of *Fol*, was used for fungal quantification (Table 1). To normalize the fungal DNA levels across different samples, the *S. lycopersicum* ubiquitin gene was used as a reference, employing the primer pair ubi3-F and ubi3-R (Rotenberg et al. 2006) (Table 1). The qPCR cycling conditions consisted of an initial denaturation at 95 °C for 3 min, followed by 40 cycles of denaturation at 95 °C for 3 s, and annealing/extension at 60 °C for 20 s. Primer specificity and the absence of primer–dimer formation was confirmed by dissociation curve analysis. A standard curve was generated using tenfold serial dilutions of the *Fol* PCR product to quantify the pathogen.

**Table 1:** Primer Sequences for Gene Expression and Fungal Quantification

Gene name	Primer name	Primer sequence (5′–3′)	Product size (bp)	References
*F. oxysporum* f. sp. *lycopersici* rDNA-IGS	sp1-2f	5′-GCTGGCGGATCTGACACTGT-3′	99	Inami et al. (2010)
	sp1-2r	5′-CCTAAACCACATATCTCGTCCAAA-3′		
*S. lycopersicum* ubiquitin (*ubi3*)	ubi3-F	5′-GCCGACTACAACATCCAGAAGG-3′	143	Rotenberg et al. (2006)
	ubi3-R	5′-TGCAACACAGCGAGCTTAACC-3′		Dimopoulou et al. (2019)
Pathogenesis-related leaf protein 6 (*PR1b1*)	pr1b1-F	5′-GGTCGGGCACGTTGCA-3′	69	Dimopoulou et al. (2019)
	pr1b1-R	5′-GATCCAGTTGCCTACAGGACATA-3′		
Ethylene response factor 1 (*ERF1)*	erf1-F	5′-TGGAGTTAGAAAGAGGCCATGG-3′	143	Dimopoulou et al. (2019)
	erf1-R	5′-CCCTCATTGATAATGCGGCTT-3′		
Enhanced diseased susceptibility 1 (*EDS1*)	eds1-F	5′-GATGCATTCAAGATTCAAAACACC-3′	96	Dimopoulou et al. (2019)
	eds1-R	5′-CAACATTTCAATGATCTCATCCCA-3′		
MYC2 transcription factor (*MYC2*)	myc2-F	5′-TAGCCACACTGGAGGCAAGAT-3′	140	Dimopoulou et al. (2019)
	myc2-R	5′-CTAGGTCTAATTCCATGAGCGC-3′		
Lipoxygenase D (*loxD*)	loxD-F	5′-CCATCCTCACCACCCTCATC-3′	139	Dimopoulou et al. (2019)
	loxD-R	5′-TACTCGGGATCGTTCTCGTC-3′		
Plant Defensin 1.2 (*PDF1.2*)	pdf1.2-F	5′-AAAAAGTGGCAAGTGGAATGG-3′	164	Dimopoulou et al. (2019)
	pdf1.2-R	5′-AATGGCAAGGTGAGTAGCAGTAA-3′		
Ascorbate Peroxidase 1 (*APX1*)	apx1-F	5′-ACTTCACGGAGCTTTTGAGTGG-3′	141	Dimopoulou et al. (2019)
	apx1-R	5′-CAGCATAGTCAGCAAAGAAGGC-3′		
Pto-interacting protein (*Pti5*)	pti5-F	5′-ATTCGCGATTCGGCTAGACAT-3′	119	Dimopoulou et al. (2019)
	pti5-R	5′-AGTAGTGCCTTAGCACCTCGCA-3′		

### In vitro* assay for antimicrobial effect of root exudates*

Root exudates were collected from tomato plants (Turrà et al. 2015) to evaluate their inhibitory effects on *Fol* in dual culture assays. Tomato seeds of landraces ARI00732 and ARI00733 were surface sterilized, planted in sterilized growth substrate (Plantobalt substrate 2, Plantaflor, Vechta, Germany) and grown in a controlled chamber as previously described. At the third leaf stage, the plants were carefully removed from the substrate and their roots were gently rinsed with sterile distilled water to remove residual particles. The plants were then transferred to sterile distilled water and maintained under controlled conditions (25 °C, 16-h light/8-h dark cycle) for 48 h to allow for root exudate release. The exudate solution was filtered through a 0.22 µm filter and stored at − 20 °C until use. To measure fresh root weight, roots were cut from individual plants, gently blotted with a paper towel and weighed.

To assess the antifungal activity of root exudates, two distinct dual culture assays were performed. In the first assay, a 5-mm mycelial plug of *Fol* was placed approximately 2 cm from the edge of a potato dextrose agar (PDA) plate. Root exudates solutions from each tomato genotype were streaked on the opposite side of the plate, also about 2 cm from the edge. Plates were incubated at 28 °C for 6 days. Fungal growth was evaluated by measuring the radial growth of *Fol* in each treatment and comparing it to control plates containing only fungal plugs. Statistical analysis of radial growth was performed to determine whether the root exudates had any effect on *Fol* development. This assay was employed to evaluate the fungistatic or fungicidal effects of root exudates on established fungal mycelium, reflecting their potential to limit pathogen spread within infected plant tissues. In the second assay, 5 ul of a conidial suspension of *Fol* (10^7^ conidia/ml) was spot-inoculated onto PDA plates pre-treated with 100 ul of root exudate solution. Plates were incubated at 28 °C for 7 days, with PDA supplemented with sterile distilled H_2_O serving as a negative control. Fungal growth was assessed as described above. This assay was used to examine the ability of root exudates to prevent fungal infection at its earliest stages by inhibiting conidial germination and early hyphal development.

### Chemotropism assay for root exudates

To evaluate the chemotropic response of *Fol* to tomato root exudates, a chemotropism assay was conducted following the protocol outlined by Turrà et al. (Turrà et al. 2015). *Fol* conidia were harvested from a liquid culture grown in Czapek-dox medium (Thermo Scientific™ Oxoid™) and diluted in 0.5% (w/v) water agar (Thermo Scientific™ Oxoid™) to a final concentration of 2.4 × 10^8^ conidia/mL. Standard Petri dishes (94 mm in diameter, with vents; Greiner Bio-One) were filled with 4 mL of this conidial suspension in water agar. Three parallel Lines, spaced 0.5 mm apart, were marked on the underside of each plate. Along the two outer Lines, square wells were cut into the agar and filled with 50 µL of either root exudate solution or sterile distilled water. Pectin [1% (w/v), Fluka Analytical] dissolved in distilled water was included as a positive control (Turrà et al. 2015). Root exudates from landraces ARI00732 and ARI00733, as well as from the susceptible cultivar Ailsa Craig, were prepared as previously described. Plates were sealed with parafilm, wrapped in aluminum foil, and incubated overnight at 28 °C. After 12 h, plates were transferred to 4 °C to halt further hyphal growth.

The orientation of *Fol* conidial germ tubes was assessed under a Zeiss Axioskop II microscope at 200 × magnification. Hyphal tips were counted as growing toward the root exudate, toward the water control, or neutral (showing no directional growth). Chemotropism was expressed as the percentage of hyphae oriented toward the root exudate or water control. For each treatment, 400–600 germinating hyphae were evaluated.

### Gene expression analysis in tomato plants following Fol infection

To analyze gene expression in tomato plants following *Fol* infection, plants were inoculated with either *Fol* or mock-inoculated with sterile distilled water as described previously. Sampling was conducted at 3 and 6 days post-inoculation (dpi), representing early and intermediate stages of the tomato - *Fol* interaction. The aboveground parts of twelve plants from each treatment group were harvested, as systemic defense responses are predominantly reflected in aerial tissues, including leaves and stems. Four plants per treatment were pooled to create each biological replicate. The plant tissues were immediately frozen in liquid nitrogen and stored at − 80 °C. Total RNA was extracted from 100 mg of tissue ground with liquid nitrogen using the NucleoZOL reagent (Macherey–Nagel, Düren, Germany) following the manufacturer's protocol. The extracted RNA was treated with DNase I (Sigma-Aldrich, St. Louis, MO, USA) to eliminate any residual genomic DNA. RNA concentrations were determined using a Nanodrop 2000 spectrophotometer (Thermo Scientific). First-strand cDNA was synthesized from 1 µg of total RNA using the Fast Gene Scriptase II cDNA 5 × ReadyMix (NIPPON Genetics Europe, Düren, Germany). Quantitative real-time PCR (qPCR) was performed under standard cycling conditions with the synthesized cDNA as a template. The expression levels of the target genes *PR1b1*, *ERF1*, *EDS1*, *MYC2*, *LoxD*, *PDF1.2*, *APX1* and *Pti5* were assessed using the primers listed in Table 1. Ubiquitin was used as a reference gene normalize expression levels. The specificity of the qPCR products was confirmed by melting curve analysis to ensure the absence of nonspecific amplification and primer dimers. Relative gene expression was calculated using the Pfaffl method (Pfaffl et al. 2002).

### Statistical analysis

All experiments were performed twice independently, each including 12 plants per treatment. Pooled samples (3 biological Replicates; 4 plants pooled per replicate) were used for gene expression and fungal quantification assays. Data were tested for normality and homogeneity of variance using the Shapiro–Wilk and Brown–Forsythe tests, respectively. Differences among treatments for AUDPC values of the first trial, plant growth parameters of the second trial and root exudates, were analyzed using one-way ANOVA, followed by Tukey’s HSD post hoc test for multiple comparisons (*P* < 0.05). In figures where letters are used to denote significance, the absence of letters above columns indicates that no significant differences were detected among treatments in that comparison. Differences between treatments for plant growth parameters of the first trial, AUDPC values of the second trial, fungal biomass quantification, and chemotropism assays were evaluated using two-tailed *t*-tests. Statistical analyses were performed using GraphPad Prism (version 8.3.0). Gene expression data were analyzed using the pairwise fixed reallocation randomization test implemented in the REST software (Pfaffl et al. 2002), with significance set at *P* < 0.05.

## Results

### Disease resistance and plant performance evaluation of Cypriot tomato landraces against Fusarium oxysporum f. sp. lycopersici

**Fig. 1:**
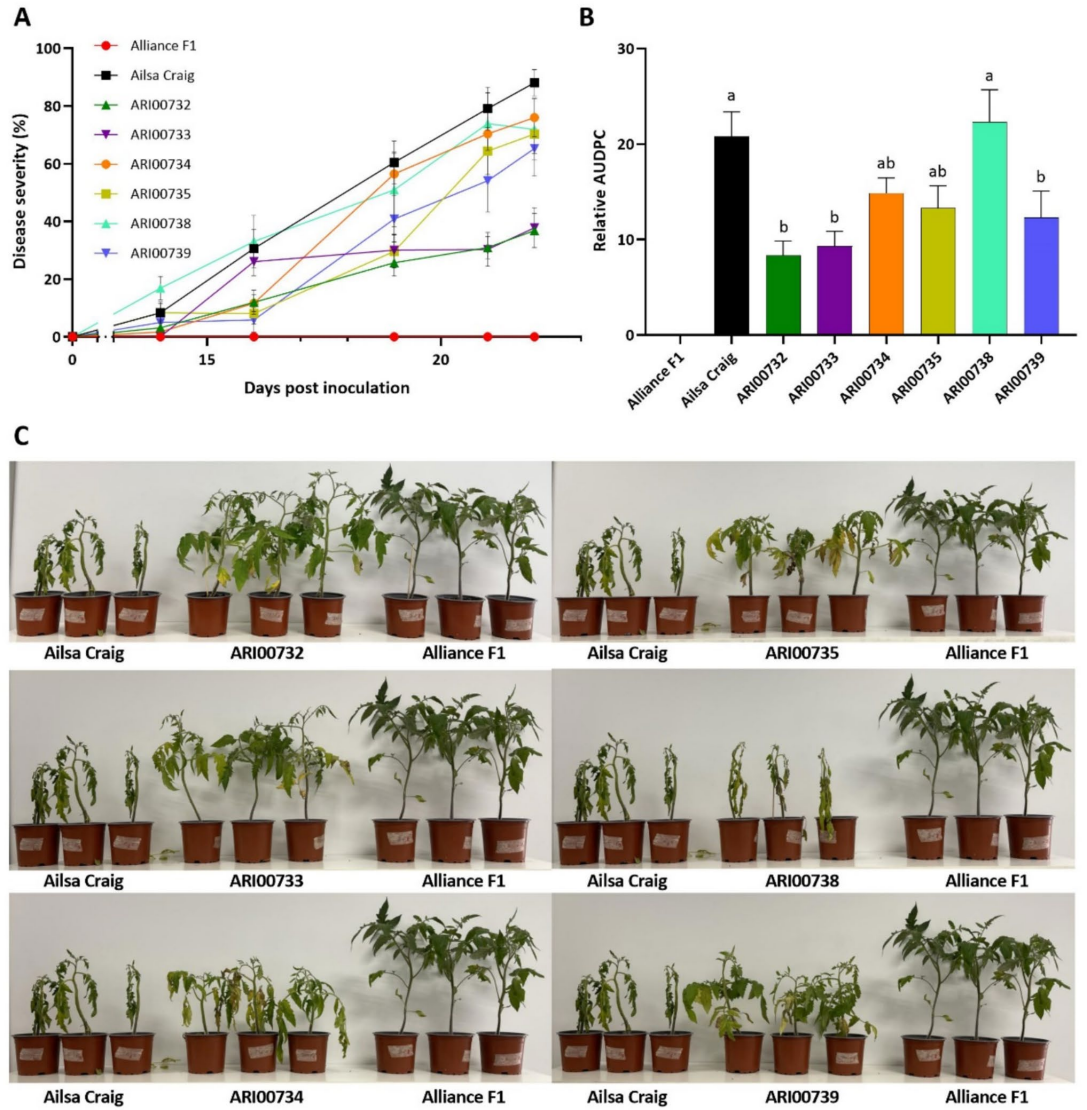
Pathogenicity experiment under controlled conditions: **a** Disease progression over time in tomato plants infected with *Fusarium oxysporum* f. sp. *lycopersici* (*Fol*); **b** amount of *Fol* disease expressed as relative AUDPC; **c** Representative images of tomato plants at 22 days post-inoculation with *Fol*. Alliance F1 plants remained asymptomatic, while ARI00734, ARI00735 and ARI00738 exhibited wilting symptoms comparable to the susceptible cultivar Ailsa Craig. In contrast, ARI00732, ARI00733 and ARI00739 displayed significantly reduced wilting, indicating partial resistance to *Fol*. Vertical bars represent the standard error (SE) of the mean (n = 12 replicates). Different letters indicate significant differences (*P* < 0.05; one-way ANOVA, Tukey’s test)

In the first experiment the disease resistance of six Cypriot tomato landraces was evaluated against *Fol* through pathogenicity experiments under controlled growth-room conditions. Initial symptoms were observed on the 14th day post-inoculation (dpi), with symptoms progressing as wilting followed by yellowing and necrosis of leaves, until 22 dpi (Fig. 1a). Disease severity progression differed among the tested genotypes over time (Fig. 1a). The susceptible control, Ailsa Craig, showed a steady increase in disease severity, reaching approximately 90% by the final assessment. In contrast, the resistant control, Alliance F1, remained complete resistant, with no visible disease symptoms throughout the experiment. Among the tested landraces, ARI00732 and ARI00733 exhibited the lowest disease severity, reaching only 37% and 38%, respectively, at the final time point. Conversely, ARI00738 developed disease severity levels similar to those of Ailsa Craig, while ARI00734, ARI00735 and ARI00739 showed intermediate disease progression patterns (Fig. 1c).

These trends were further supported by the relative AUDPC values and post-hoc comparisons (Tukey’s HSD), which revealed distinct resistance profiles among the Cypriot tomato landraces and controls (Fig. 1b). Ailsa Craig and the landrace ARI00738 exhibited the highest relative AUDPC values (20.77% and 22.33% respectively), while the resistant control (Alliance F1) showed no disease progression, confirming by its resistance the experimental validity. ARI00732, ARI00733 and ARI00739 displayed the strongest resistance, with significantly lower relative AUDPC values (8.36%, 9.30%, and 12.30%, respectively) compared to Ailsa Craig and ARI00738. ARI00734 (14.87%) and ARI00735 (13.30%) exhibited intermediate resistance, showing statistical overlap with both resistant and susceptible landraces, indicating variability in their resistance responses.

**Fig. 2:**
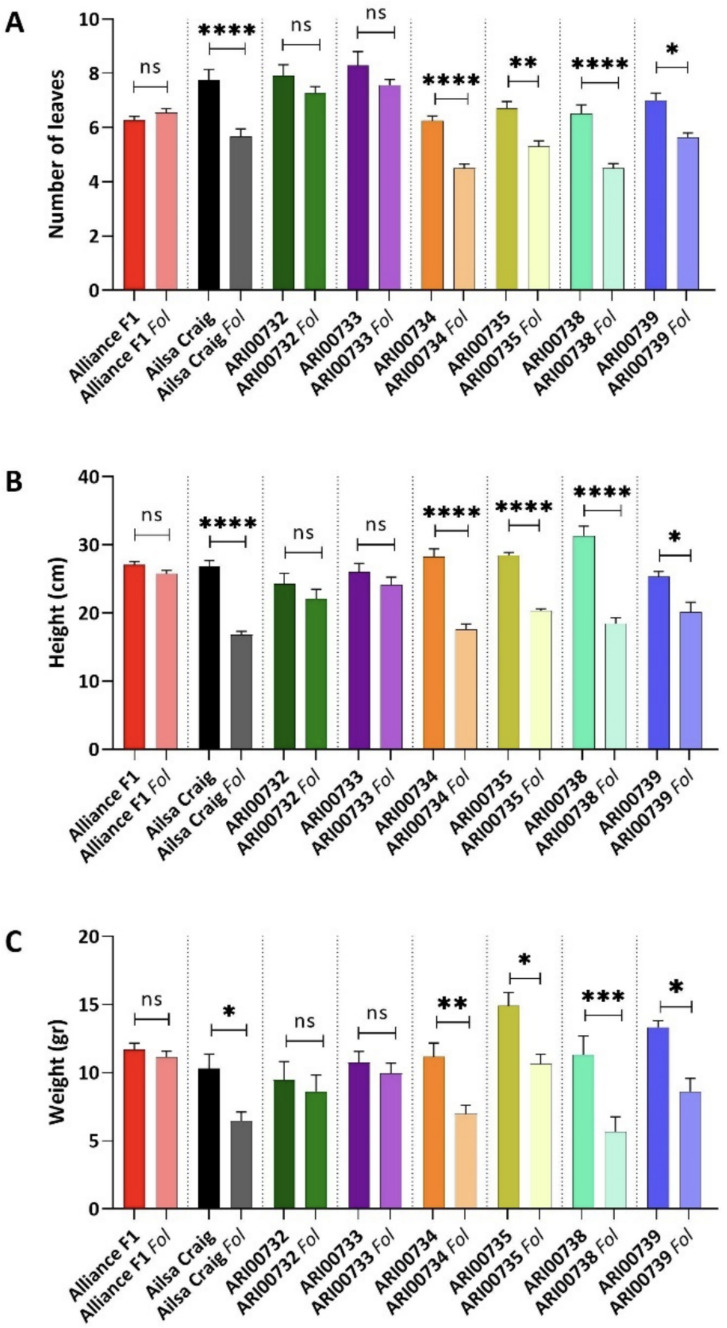
Impact of *Fusarium oxysporum* f. sp. *lycopersici* (*Fol*) infection on plant growth parameters: **a** Number of leaves, **b** plant height and **c** plant fresh weight measured in infected and non-infected tomato genotypes. Comparisons are made within each genotype between inoculated and uninoculated plants. *Fol* infection significantly reduced all growth parameters in the susceptible control Ailsa Craig and the landraces ARI00734, ARI00735, ARI00738 and ARI00739 (*P* ≤ 0.0001–0.05). In contrast, the resistant control Alliance F1, along with ARI00732 and ARI00733, maintained similar values between infected and non-infected plants, suggesting a stronger ability to sustain vegetative growth under pathogen pressure. Vertical bars represent the standard error (SE) of the mean (n = 12 replicates). Asterisks indicate statistically significant differences between inoculated and uninoculated plants within each genotype: *P* < 0.05 (*), *P* < 0.01 (**), *P* < 0.001 (***), *P* < 0.0001 (****) (two-tailed *t*-test)

To further assess the impact of *Fol* infection on plant performance, we measured the number of leaves, plant height, and plant weight across the tomato genotypes under study. Significant differences were observed between infected and non-infected plants, with variation in susceptibility among the tested genotypes (Fig. 2). Regarding the number of leaves, *Fol* infection significantly reduced leaf count in the susceptible control Ailsa Craig (*p* ≤ *0.0001*), as well as in ARI00734, ARI00735, ARI00738 and ARI00739 (*p* ≤ *0.0001—0.05*). In contrast, the resistant control, Alliance F1, along with ARI00732 and ARI00733, showed no significant reduction in leaf number, indicating their ability to maintain vegetative growth despite pathogen infection (Fig. 2a). Similarly, *Fol* infection led to a significant reduction in plant height in Ailsa Craig (*p* ≤ *0.0001*) and in the same set of landraces (ARI00734, ARI00735, ARI00738 and ARI00739; *p* ≤ *0.0001–0.05*). The resistant control, Alliance F1, along with ARI00732 and ARI00733, maintained comparable heights between infected and non-infected plants, further supporting their resistance to *Fol* (Fig. 2b). Weight measurements followed the same trend, with *Fol* infection significantly reducing biomass in Ailsa Craig and the susceptible landraces ARI00734, ARI00735, ARI00738 and ARI00739 (*p* ≤ *0.001–0.05*). In contrast, Alliance F1, ARI00732 and ARI00733 maintained similar weights between infected and non-infected plants, suggesting a stronger ability to sustain growth under pathogen pressure (Fig. 2c).

Overall, these results indicate that ARI00732 and ARI00733 exhibit higher resistance than the susceptible cultivar, though not to the extent of the resistant control, while ARI00734, ARI00735 and ARI00738 demonstrate greater susceptibility, aligning with previous disease severity and AUDPC assessments. Similarly, ARI00739 showed increased resistance compared to the susceptible control; however, it remained susceptible to pathogen-induced growth suppression.

### Effect of soil sterilization on disease severity and growth parameters

To assess whether the resistance observed in ARI00732 and ARI00733 was affected by the presence of beneficial microorganisms in the soil, a second experiment was conducted comparing plants grown in sterile and non-sterile soil.

**Fig. 3:**
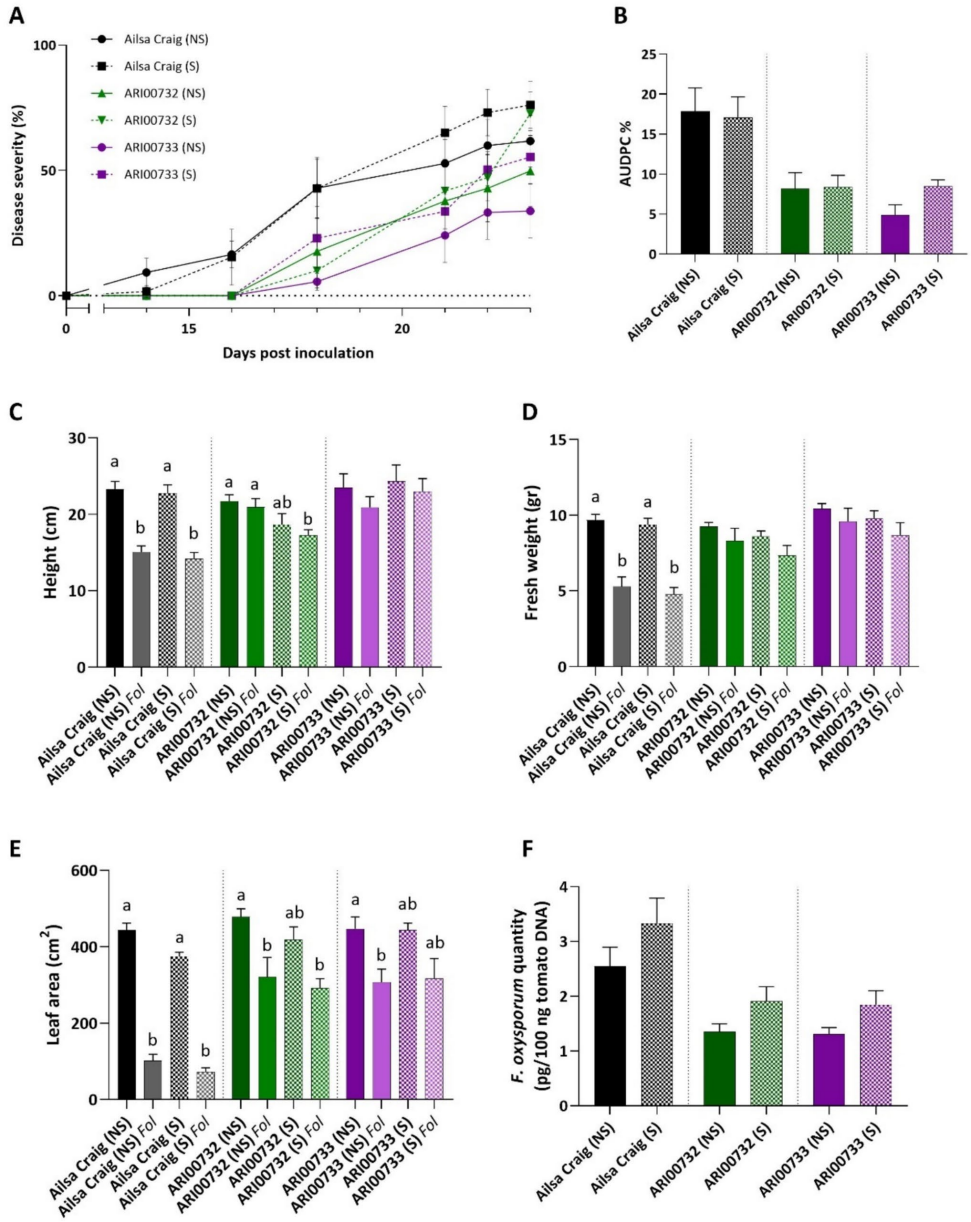
Effect of soil sterilization on *Fusarium oxysporum* f. sp. *lycopersici* (*Fol*) disease severity and growth parameters: **a** Disease progression in tomato plants infected with *Fol* under sterile (S) and non-sterile (NS) soil conditions, **b** relative AUDPC values comparing all genotypes under sterile and non-sterile conditions, **c–e** plant growth parameters—height (**c**), fresh weight (**d**) and leaf area (**e**)—for inoculated and uninoculated plants, with statistical comparisons conducted within each genotype under sterile (S) and non-sterile (NS) soil conditions, **f** *Fol* biomass in aboveground tissues at 23 dpi, quantified by qPCR using total plant DNA, comparing all genotypes under sterile (S) and non-sterile (NS) conditions. Vertical bars represent the standard error (SE) of the mean. Different letters above columns indicate significant differences (*P* < 0.05, one-way ANOVA, Tukey’s HSD test), while the absence of letters denotes no significant differences among treatments in that panel

Disease severity progression followed a similar trend across both soil conditions, with the susceptible control, Ailsa Craig, exhibiting the highest disease severity levels. Initial symptoms were observed at 14 dpi, with disease severity increasing until the final assessment at 23 dpi (Fig. 3a). The non-sterile and sterile soil conditions had no significant effect on disease progression in Ailsa Craig, as both treatments reached similar disease severity levels (Fig. 3b). In contrast, ARI00732 and ARI00733 consistently exhibited lower disease severity compared to Ailsa Craig, in both treatments (Fig. S1a, b). Notably, ARI00733 plants grown in non-sterile soil showed slightly lower disease severity over time, suggesting a potential contribution of soil microbiota to disease suppression although no statistical differences were observed in AUDPC values (Fig. 3b). Ailsa Craig had the highest AUDPC values under both soil conditions (Fig. S1a, b), with no significant differences between sterile and non-sterile treatments (Fig. 3b). In contrast, ARI00732 and ARI00733 exhibited significantly lower AUDPC values compared to Ailsa Craig (*P* ≤ 0.05) (Fig. S1a, b), with no statistically significant differences between sterilized and non-sterilized conditions (Fig. 3b). This indicates that while these landraces possess intrinsic resistance to *Fol*, the presence of soil microbiota may provide additional, albeit limited, disease suppression.

In terms of plant growth parameters, *Fol* infection significantly reduced plant height, fresh weight and leaf area in Ailsa Craig under both soil conditions (Fig. 3c, d and e). In contrast, ARI00732 and ARI00733 exhibited only slight reductions in height and fresh weight upon infection, but these differences were not statistically significant. Similar to Ailsa Craig, infected plants of ARI00732 and ARI00733 showed a decrease in leaf area. However, the reduction was more pronounced in Ailsa Craig. While plants grown in non-sterile soil maintained slightly higher fresh weight and leaf area, the differences were not statistically significant, suggesting that soil microbiota had no substantial effect in mitigating pathogen-induced growth suppression.

Overall, these findings indicate that the resistance observed in ARI00732 and ARI00733 is primarily determined by their genetic background rather than by the influence of soil microbiota. Although beneficial microorganisms in non-sterile soil may contribute slightly to disease suppression, they did not significantly affect disease severity or plant growth parameters under the conditions tested. This highlights that intrinsic resistance mechanisms in these landraces are the dominant factor in their defense against *Fol* infection.

### Fungal quantification

In an effort to determine whether the reduced disease severity observed in the ARI00732 and ARI00733 landraces is associated with lower fungal biomass in plant tissues, the level of *Fol* colonization in the vascular tissues of tomato plants was quantified using real-time PCR. The results showed that *Fol* DNA levels were significantly higher in Ailsa Craig plants than in the landraces ARI00732 and ARI00733, regardless of whether they were grown in non-sterile or sterile substrate (Fig. S1c, d). Specifically, when plants were grown in non-sterile substrate, fungal DNA levels in Ailsa Craig were approximately two-fold higher than in ARI00732 and ARI00733 (Fig. S1c). Similarly, when plants were grown in sterile substrate, fungal DNA levels in Ailsa Craig were about 1.8 times higher than in the resistant landraces (Fig. S1d). Additionally, within each genotype, no significant differences in fungal biomass were observed between plants grown in non-sterile and sterile conditions, indicating that soil microorganisms were not a determining factor in reducing pathogen colonization (Fig. 3f). This finding aligns with the pathogenicity experiment results, which showed no significant protection conferred by soil microorganisms, as plants grown in non-sterile substrate did not exhibit enhanced resistance to *Fol* infection. Therefore, the resistance observed in ARI00732 and ARI00733 landraces is not attributed to soil microorganisms but is more likely due to their genetic background.

### Effect of root exudates on Fusarium oxysporum f. sp. lycopersici growth and chemotropism

Following the pathogenicity experiments, we wanted to investigate whether the resistance observed in landraces ARI00732 and ARI00733 could be attributed to the composition of their root exudates. Specifically, we aimed to determine whether these exudates exhibited antifungal activity against *Fol* and whether they affected the directional growth of the pathogen through negative chemotropism.

**Fig. 4:**
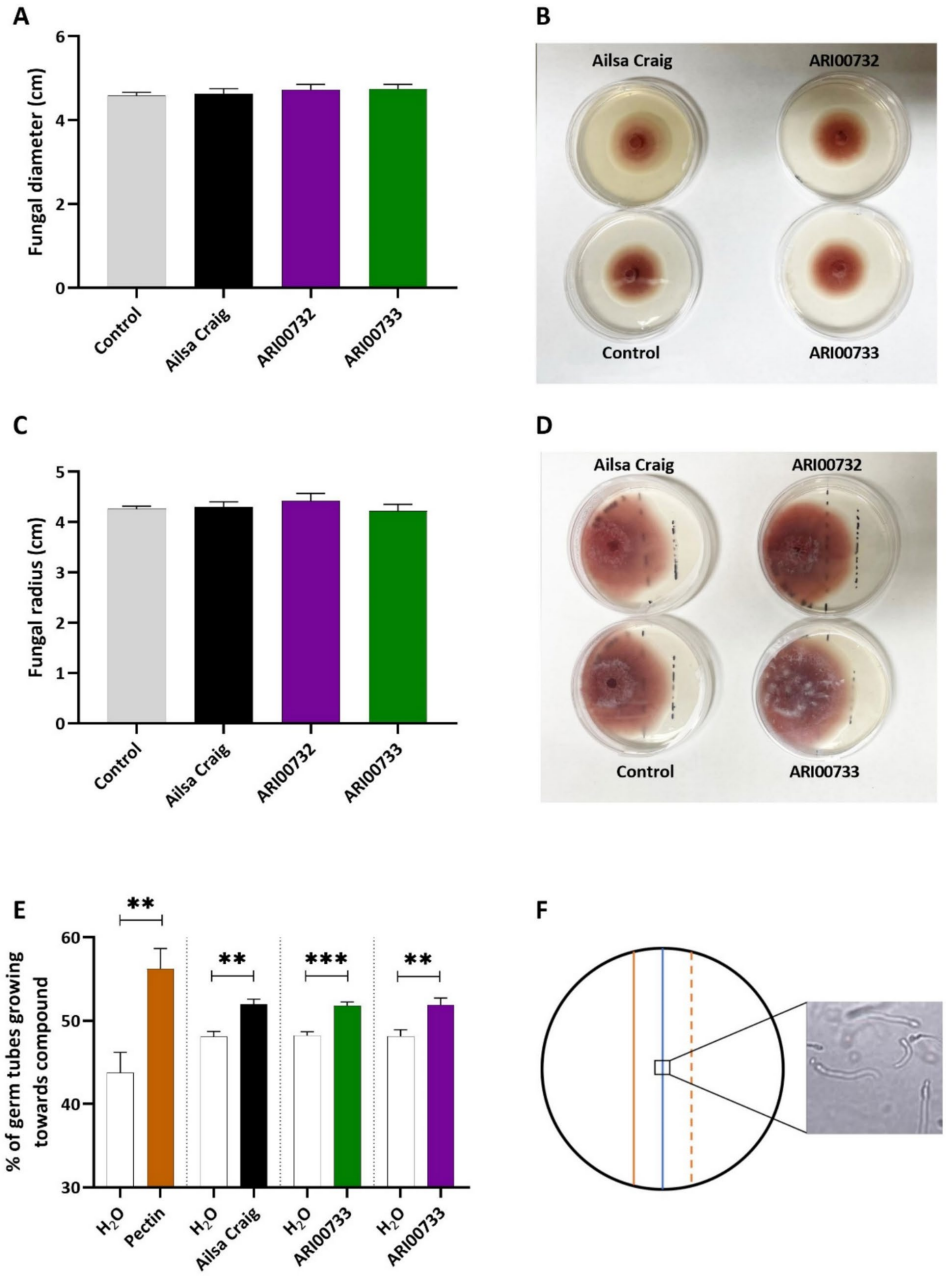
Effect of tomato root exudates on *Fusarium oxysporum* f. sp. *lycopersici* (*Fol*) growth and chemotropism: **a** fungal growth diameter on PDA plates pre-treated with root exudates from Ailsa Craig, ARI00732, and ARI00733, compared to untreated control plates, **b** representative images of *Fol* growth on PDA plates pre-treated with tomato root exudates, **c** fungal growth radius on PDA plates where root exudates were streaked ~ 2 cm from the plate edge, **d** representative images of *Fol* growth on PDA plates with root exudates streaked 2 cm from the plate edge, **e** chemotropism assay showing directional hyphal growth of *Fol* in response to root exudates or pectin (positive control). Dashed vertical lines separate the different combinations tested. In each combination, the percentages of spores attracted by either the solvent or the tested compound add up to 100%. Shown are the mean percentages of three replicates per treatment, each based on hyphal orientation counts of ca. 400 – 600 germinating conidia. Vertical bars represent the standard error (SE) of the mean. Asterisks indicate statistically significant differences within each treatment combination: *P* < 0.01 (**), *P* < 0.001 (***) (two-tailed *t*-test). **f** Representation of the chemotropism test in which the orientation of hyphal growth was tested on the blue line in the middle, with the water on the red line and the test root exudates or pectin on the dashed line. The photograph is a representative image of germinating *Fol* spores at the moment of scoring

The fungal growth diameter on the plates containing root exudates from the resistant landraces and the susceptible cultivar Ailsa Craig did not differ significantly from the control plates. In the experiments where the exudates were plated on the surface of the medium, the fungal diameter was 4.58 cm for the control plates, 4.62 cm for the plates with Ailsa Craig exudates, 4.72 cm for the plates with ARI00732 exudates, and 4.74 cm for the plates with ARI00733 exudates (Fig. 4a, b). Similarly, in the experiments where the root exudates were streaked in a rod shape approximately 2 cm from the edge of the plate, the fungal radius was 4.26 cm for the control plates, 4.30 cm for the plates with Ailsa Craig exudates, 4.42 cm for the plates with ARI00732 exudates and 4.22 cm for the plates with ARI00733 exudates (Fig. 4c, d).

To assess the effect of root exudates on *Fol* hyphal growth, we performed a chemotropism assay based on the method developed by Turrà et al. (2015). Root exudates from the landraces ARI00732, ARI00733 and the Ailsa Craig cultivar were tested, with pectin serving as a positive control. Fungal hyphae from germinating conidia were chemotropically attracted toward pectin, confirming previous findings (Turrà et al. 2015; Stringlis et al. 2018) while root exudates from all tomato genotypes significantly attracted the fungal hyphae (Fig. 4e, f). Together, these results indicate that the observed resistance in landraces ARI00732 and ARI00733 is unlikely to be attributed to the ability of their root exudates to repel or inhibit the pathogen through chemotropism.

### Gene expression analysis

**Fig. 5:**
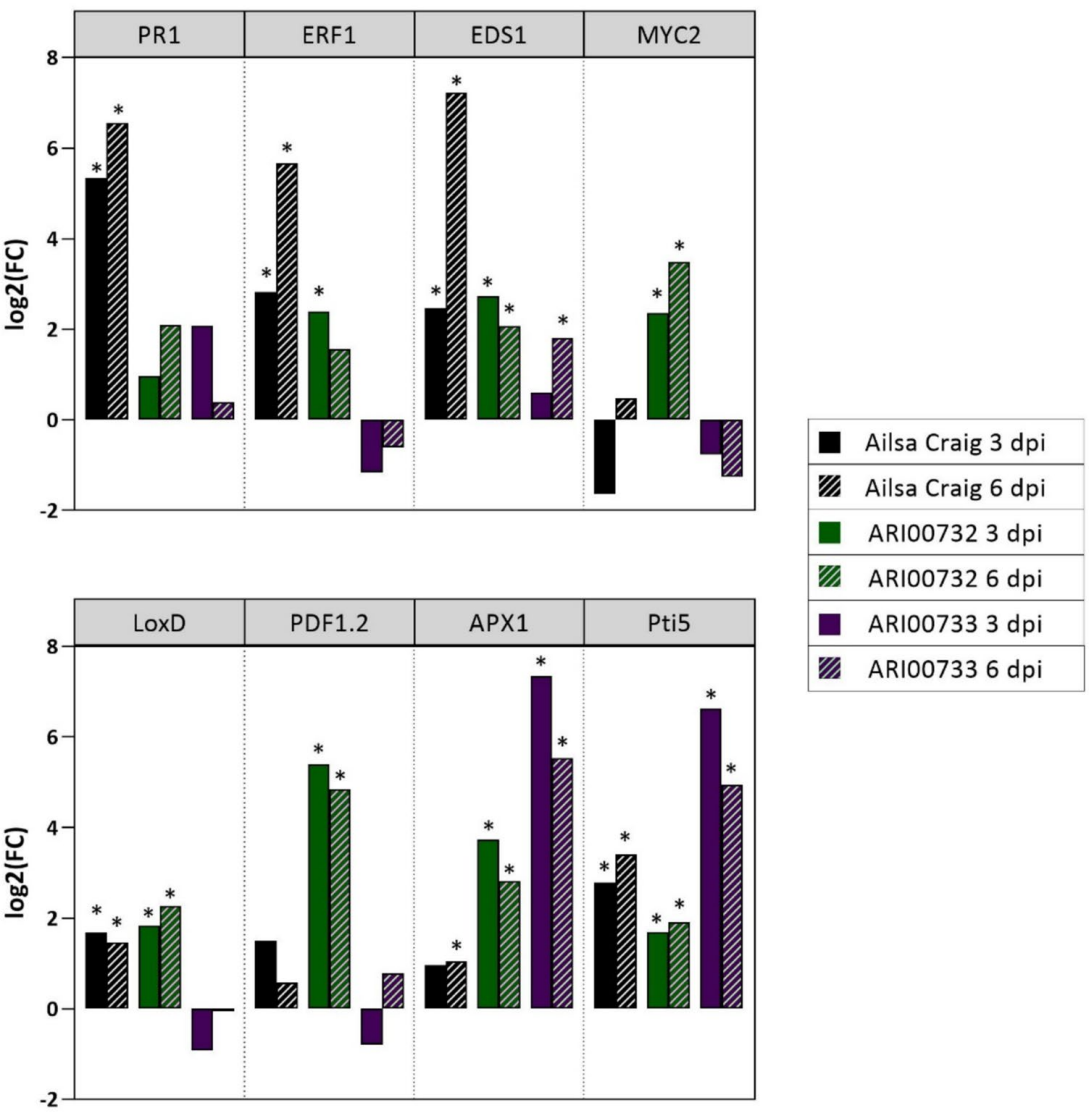
Effect of *Fusarium oxysporum* f. sp. *lycopersici* (*Fol*) inoculation on the expression of defense-related genes. Relative expression (log_2_ fold change) of selected defense genes (*PR1*, *ERF1*, *EDS1*, *MYC2*, *LoxD*, *PDF1.2*, *APX1*, *Pti5*) in tomato genotypes Ailsa Craig, ARI00732, and ARI00733 at 3- and 6-days post-inoculation (dpi) with *Fol*. Gene expression was normalized to the reference gene *UBI3.* Fold change (FC) values were calculated using REST-XL (version 2) and represent the ratio of expression in *Fol*-inoculated plants relative to mock-inoculated (water-treated) controls. Asterisks indicate statistically significant differences compared to the respective mock controls (*p* < 0.05, pairwise fixed reallocation randomization test, REST-XL)

To investigate the molecular mechanisms underlying the differential resistance of ARI00732 and ARI00733 landraces to *Fol* infection, the expression levels of defense-related genes were analyzed at 3- and 6-days post-inoculation (dpi) using qRT-PCR. The results are presented as log_2_ fold-change (FC) relative to the mock-treated plants (Fig. 5).

The expression of *PR1*, *ERF1* and *EDS1*, key markers of the salicylic acid (SA) and ethylene (ET) pathways, was strongly induced in Ailsa Craig particularly at 6 dpi, showing the highest upregulation among the genotypes. In contrast, ARI00732 and ARI00733 showed no significant induction of *PR1* and *ERF1* genes, except for a moderate *ERF1* upregulation in ARI00732 at 3 dpi. *EDS1* was moderately induced in both landraces, although at lower levels compared to Ailsa Craig. *MYC2*, a key regulator of the jasmonic acid (JA) pathway, was significantly upregulated in ARI00732 at both time points, whereas its expression was not induced in Ailsa Craig and ARI00733. *LoxD* and *PDF1.2* were significantly upregulated in ARI00732, indicating a strong activation of JA-mediated defense responses. In contrast, ARI00733 showed no induction of these genes. *LoxD* was also upregulated in Ailsa Craig at both time points, but to a lesser extent than in ARI00732. The antioxidant defense-related gene *APX1* was highly expressed in both ARI00732 and ARI00733, with ARI00733 exhibiting the strongest induction. Similarly, *Pti5*, a transcription factor involved in SA-dependent defense, was highly induced in ARI00733 at both time points, whereas its expression in Ailsa Craig and ARI00732 was moderate.

These findings suggest distinct defense strategies among the genotypes. Ailsa Craig primarily relies on SA- and ET-mediated responses, particularly at later stages of infection. ARI00732 exhibits a strong JA-dependent defense, while ARI00733 combines antioxidant and SA-mediated responses.

## Discussion

Local tomato populations are increasingly recognized as valuable reservoirs of genetic resistance against plant pathogens (Lazaridi et al. 2024), including *Fusarium oxysporum* f. sp. *lycopersici* (*Fol*), the causal agent of Fusarium wilt (Kawicha et al. 2023). This soilborne disease poses a major threat to global tomato production, causing severe yield losses under optimal conditions for pathogen development (McGovern 2015b; Nirmaladevi et al. 2016; Gaber et al. 2025). Given the limitations of chemical control and the breakdown of resistance in commercial tomato cultivars due to the emergence of new *Fol* races, there is a growing need to identify new genetic sources of resistance.

In this study, we evaluated the resistance of six Cypriot tomato landraces against *Fol*, focusing on disease severity and plant performance under disease, pathogen colonization, root exudate effects, and defense-related gene expression. Our findings revealed that two landraces, ARI00732 and ARI00733, exhibited significantly lower disease severity compared to the susceptible control, Ailsa Craig, suggesting the presence of effective resistance mechanisms. To further elucidate the basis of their resistance, we examined whether it was affected by soil microbiota, the antifungal activity of root exudates, or plant defense gene activation.

To assess the potential role of beneficial soil microorganisms in the resistance of ARI00732 and ARI00733, we conducted pathogenicity experiments under both sterile and non-sterile soil conditions. The absence of significant differences in disease severity between these treatments suggests that beneficial soil microbiota did not contribute substantially to disease suppression. Instead, our results indicate that these landraces possess intrinsic resistance mechanisms that limit pathogen colonization. This finding is consistent with previous studies showing that resistance to *Fol* is primarily conferred by host genetic factors (Chitwood-Brown et al. 2021; Kawicha et al. 2023). However, our results differ from studies suggesting that soil microbiota can enhance resistance to *Fol*. Antoniou et al. (2017) reported that beneficial rhizosphere microbes can prime plant immune responses, thereby reducing pathogen colonization. Similarly, Chialva et al. (2018) demonstrated that soil microbiomes can induce a state of alert in tomato plants, activating oxidative stress responses, phenol biosynthesis, lignin deposition and innate immunity, ultimately enhancing resistance to *Fol*. Additionally, Tsolakidou et al. (2019) highlighted the role of specific microbial communities in suppressing *Fol* infection. In contrast, our findings indicate that the resistance observed in ARI00732 and ARI00733 is not microbiome-dependent, as plants grown in non-sterile soil did not exhibit enhanced resistance compared to those in sterile conditions. This discrepancy may be attributed to differences in soil microbial composition, as the suppressive potential of microbiota varies depending on environmental conditions, soil type and plant genotype. Further investigations into the rhizosphere microbiome of these landraces could help clarify whether specific microbial communities contribute to resistance under field conditions.

We then aimed to determine whether root exudates from the resistant landraces ARI00732 and ARI00733 exhibited antifungal activity against *Fol* compared to the susceptible cultivar Ailsa Craig. Our results showed that root exudates from both resistant and susceptible genotypes did not inhibit *Fol* growth, suggesting that resistance in ARI00732 and ARI00733 is not mediated by the secretion of antifungal compounds or pathogen-deterrent metabolites. Instead, the observed resistance is likely driven by internal plant defense responses. Direct evidence supporting antifungal activity of tomato root exudates against *Fol* remains limited in the literature. However, research suggests that tomato root exudates play a crucial role in shaping interactions with *Fusarium oxysporum*. Steinkellner et al. (2005) found that tomato root exudates generally promote *F. oxysporum* microconidia germination, indicating that exudates may provide signals or nutrients essential for fungal activation. Interestingly, when these exudates were treated with polyvinylpolypyrrolidone (PVPP) to remove phenolic compounds, *F. oxysporum* microconidia germination increased significantly. This suggests that while tomato root exudates contain compounds that promote fungal growth, they also include phenolic compounds with inhibitory effects on spore germination. Furthermore, different *F. oxysporum* strains exhibited variable responses to tomato root exudates, highlighting the complexity of host–pathogen interactions and the potential for strain-specific adaptations to root-derived chemical cues. Our findings align with these observations, as the lack of direct antifungal activity in root exudates from ARI00732 and ARI00733 suggests that resistance in these landraces is not mediated by chemical suppression of *Fol* in the rhizosphere. Instead, their resistance likely depends on plant-mediated defense responses that are triggered following pathogen infection.

Moreover, the absence of negative chemotropism in *Fol* hyphae toward root exudates from resistant landraces further supports the notion that resistance observed in our study is not associated with root-secreted deterrent compounds but rather with systemic plant immunity. This finding is consistent with the study by Turrà et al. (2015), which demonstrated that *F. oxysporum* uses the Ste2 sex pheromone receptor to detect host-derived signals, allowing the pathogen to actively move toward root exudates rather than avoiding them. Given that *F. oxysporum* perceives host signals rather than displaying avoidance behavior, it is likely that resistant landraces do not interfere with pathogen chemotropic sensing but instead restrict infection through defense mechanisms upon pathogen infection. Further studies on the biochemical composition of root exudates from these landraces could help determine whether specific metabolites contribute to resistance through indirect mechanisms, such as priming plant defense responses or modulating microbial communities in the rhizosphere. In addition, investigating whether resistant landraces influence fungal signaling pathways, rather than directly inhibiting fungal growth, could provide new insights into host–pathogen interactions at the early stages of infection.

Since our findings indicated that resistance in ARI00732 and ARI00733 is not influenced by soil microbiota or root exudate antifungal activity, we next examined whether plant defense gene activation contributes to the observed resistance. Gene expression analysis revealed that these landraces exhibited distinct immune responses compared to the susceptible cultivar Ailsa Craig, suggesting that their resistance is driven by differential activation of defense signaling pathways. ARI00732 exhibited strong upregulation of JA-related genes (*MYC2*, *LoxD*, *PDF1.2*), indicating that JA signaling plays a central role in its defense response against *Fol*. This aligns with previous findings demonstrating that JA-mediated responses are crucial for restricting *Fusarium oxysporum* and reducing disease incidence in tomato and Arabidopsis plants (Berrocal-Lobo et al. 2002; Anderson et al. 2004; McGrath et al. 2005; Thatcher et al. 2012; Jogaiah et al. 2018; Fujikawa et al. 2021). However, some *F. oxysporum* isolates have evolved mechanisms to exploit JA signaling to promote disease progression. For instance, in Arabidopsis, COI1-mediated JA signaling has been linked to increased susceptibility, with *coi1* mutants displaying enhanced resistance (Thatcher et al. 2009). In contrast, *Fol* does not appear to exploit JA signaling in the same manner, as this forma specialis does not produce jasmonates and does not rely on the tomato COI1 homolog for pathogenicity (Cole et al. 2014). On the other hand, ARI00733 exhibited strong upregulation of *Pti5*, a transcription factor linked to SA-dependent resistance, and the *APX1*, an antioxidant defense gene, suggesting that oxidative stress regulation plays a role in its defense strategy. Enhanced *APX1* activity is typically associated with better oxidative stress management during pathogen attack, supporting sustained cellular metabolism and defense. While other defense responses have documented roles in tomato resistance to *F. oxysporum*, APX1 and other antioxidant enzymes coordinate to manage ROS during infection (Hernández-Aparicio et al. 2021). Notably, there is no direct evidence that *APX1* alone confers complete resistance to *F. oxysporum*, but studies on other plant-pathogen systems indicate that *APX1* is part of a complex network controlling the redox environment, which is essential for defense gene activation and limiting pathogen advancement (Davletova et al. 2005; Jiang et al. 2016; Qi et al. 2023). SA plays a pivotal role in plant defense mechanisms against *F. oxysporum*, primarily by inducing systemic resistance. Exogenous application of SA has been demonstrated to enhance resistance in various plant species. For instance, in tomato plants, SA treatment increased endogenous SA levels in roots and enhanced phenylalanine ammonia lyase and peroxidase activities, leading to enhanced resistance against *Fol* (Mandal et al. 2009). Similarly, in Arabidopsis, SA application activated key defense genes such as *PR1* and *BGL2*, reducing foliar necrosis and disease severity (Edgar et al. 2006). Furthermore, overexpression of Arabidopsis *NPR1* in tomato reduced *Fol* disease symptoms and stem colonization by the pathogen, further supporting the role of SA in defense (Lin et al. 2004).

Although SA and JA are often considered antagonistic, emerging evidence suggests that these phytohormones can act synergistically to enhance resistance against *Fusarium oxysporum* infections. This crosstalk between SA and JA can lead to a more robust and coordinated immune response, allowing plants to counteract different stages of *F. oxysporum* infection more effectively. For example, Zehra et al. (2017) demonstrated that the combined application of *Trichoderma harzianum*, SA and methyl jasmonate (MeJA) significantly strengthened the antioxidant defense system in tomato plants, resulting in increased resistance to *Fol*. This synergistic treatment led to higher antioxidant enzyme activity, reduced lipid peroxidation, and increased ascorbic acid levels, all contributing to disease resistance. Similarly, Jogaiah et al. (2018) found that inoculation of two-week-old tomato seedlings with *Trichoderma virens* spores activated both JA- and SA-dependent pathways, leading to resistance against *Fol*. This was evident from the upregulation of *PDF1* (JA-responsive) and *PR1a* (SA-responsive) genes in wild-type tomato plants. In contrast, mutants deficient in JA (*def1*) or SA (*NahG*) exhibited increased susceptibility to *Fol*, highlighting the necessity of both signaling pathways for effective resistance. Additionally, the cooperative role of SA and JA in defense against *F. oxysporum* has been linked to enhanced production of lignin and phenylpropanoid-related genes, which contribute to structural barriers that limit pathogen invasion (Akram et al. 2021). Collectively, these studies underscore the importance of SA-JA crosstalk in orchestrating an effective and coordinated defense mechanism against *F. oxysporum*. By leveraging the synergistic effects of these pathways, plants can enhance their resistance to such pathogenic threats.

In contrast, Ailsa Craig exhibited a delayed activation of SA/ET-related genes (*PR1*, *ERF1*, *EDS1*), which may have been insufficient to restrict pathogen colonization. Since SA-mediated defenses are primarily effective against biotrophic and hemibiotrophic pathogens and less effective against necrotrophic pathogens (Glazebrook 2005), this delayed response may have been insufficient to contain *Fol* infection. *Fol* is classified as a hemibiotrophic fungus (Srivastava et al. 2024) meaning that it initially behaves as a biotroph, maintaining host cell viability, before transitioning into a necrotrophic phase, where it induces host cell death to facilitate nutrient acquisition. This biphasic lifestyle may explain why ARI00732 and ARI00733 activated both SA- and JA-responsive genes upon infection. The strong JA-mediated response in ARI00732 and the combined SA and redox-related responses in ARI00733 suggest that these landraces mount a more comprehensive immune response, allowing them to counteract both the biotrophic and necrotrophic stages of *Fol* infection.

The identification of ARI00732 and ARI00733 as partially resistant landraces has important implications for breeding programs aimed at improving tomato resistance to Fusarium wilt. Their ability to limit disease severity and restrict pathogen colonization compared to the susceptible control suggests that they possess valuable resistance traits that could be incorporated into commercial breeding lines. Unlike traditional resistance breeding that relies on single resistance (*R*) genes, which can be rapidly overcome by evolving pathogen races, the polygenic nature of resistance in these landraces may offer a more durable and broad-spectrum defense against *F. oxysporum*. Nevertheless, the contribution of classical R genes cannot be excluded, as these have historically provided effective but race-specific resistance to *Fol*. Combining potential R-gene-mediated resistance with polygenic defense responses could enhance both durability and breadth of resistance in future breeding programs. Furthermore, the differential activation of JA- and SA-related pathways in ARI00732 and ARI00733 suggests that combining these resistance traits could enhance defense effectiveness. Future breeding efforts could explore pyramiding these traits to develop tomato cultivars with improved resistance to Fusarium wilt. Additionally, while our findings suggest that soil microbiota did not significantly influence resistance, further research could investigate whether specific microbial consortia may enhance the natural resistance of these landraces under agricultural conditions.

This study provides valuable insights into the resistance mechanisms of Cypriot tomato landraces against *Fol*, highlighting their potential as a resource for breeding programs. By focusing on two landraces with partial resistance, we were able to uncover key defense responses and lay the groundwork for exploring the genetic basis of this trait. The integration of gene expression analysis has shed light on important molecular pathways involved in resistance, offering a strong platform for future investigations. Expanding this research to include a wider range of landraces, as well as complementary omics approaches such as transcriptomics and metabolomics, could further refine our understanding of resistance variability and mechanisms. Moreover, translating these findings to field conditions will be a crucial next step to evaluate the performance of these resistance traits under natural infection pressures and diverse environments.

## Conclusion

This study provides novel insights into the resistance mechanisms of local Cypriot tomato landraces against *Fusarium oxysporum* f. sp. *lycopersici*. Our findings demonstrate that ARI00732 and ARI00733 possess intrinsic resistance, which is independent of soil microbiota or root exudate-mediated effects. Instead, their resistance appears to be driven by distinct immune signaling pathways, with ARI00732 relying on JA-mediated defenses and ARI00733 integrating SA and antioxidant responses. These findings highlight the potential of these landraces as valuable genetic resources for developing tomato cultivars with improved resistance to Fusarium wilt. Future research should focus on identifying the genetic basis of their resistance, assessing their durability under field conditions, and exploring potential synergies with beneficial soil microbes. Such efforts could contribute to more sustainable disease management strategies and the development of tomato varieties with enhanced resilience against *F. oxysporum*.

## Supplementary Information

Below is the link to the electronic supplementary material.

Supplementary file1 (DOCX 254 KB)

## Data Availability

All data generated or analyzed during this study are included in this published article.
