# Supplementary material for: Distinct immune responses confer partial resistance to Fusarium wilt in tomato landraces
Source: Planta. 2025 Sep 8;262(4):99. doi: 10.1007/s00425-025-04818-7 (PMC12417260; doi:10.1007/s00425-025-04818-7)
Supplement: Supplementary file 1 — Supplementary file1 (DOCX 254 KB) [file 425_2025_4818_MOESM1_ESM.docx]

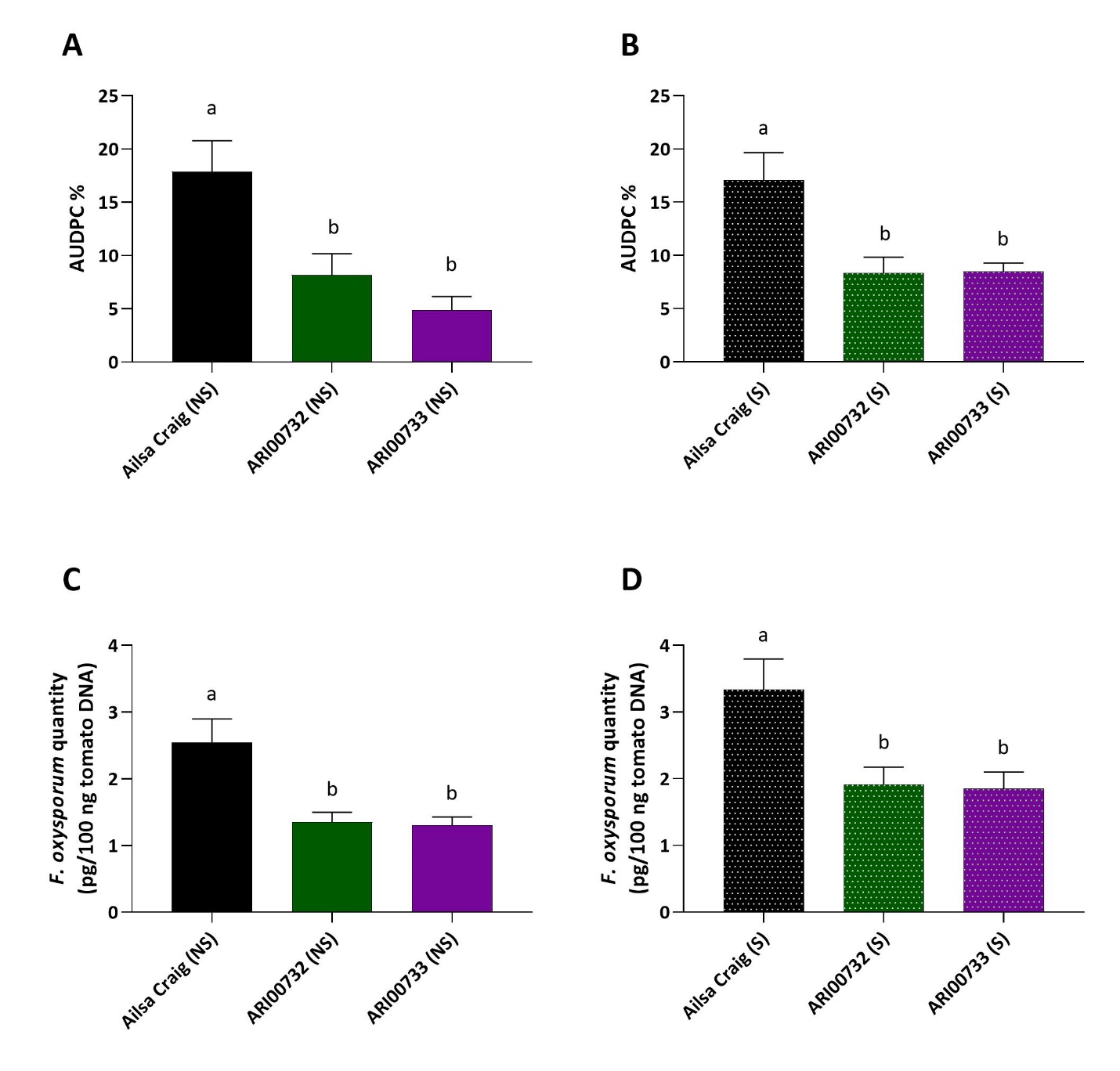


**Fig. S1** Relative AUDPC values comparing all genotypes under non-sterile **(a)** and sterile conditions **(b)**, *Fol* biomass in aboveground tissues at 23 dpi, quantified by qPCR using total plant DNA, comparing all genotypes under non-sterile **(c)** and sterile conditions **(d)**. Vertical bars represent the standard error (SE) of the mean. Different letters above columns indicate significant differences (*P <* 0.05, one-way ANOVA, Tukey’s HSD test)
